# Supplementary material for: Natural History of Marburg Virus Infection to Support Medical Countermeasure Development
Source: Viruses. 2022 Oct 18;14(10):2291. doi: 10.3390/v14102291 (PMC9607268; doi:10.3390/v14102291)
Supplement: Supplementary file 1 [file viruses-14-02291-s001.zip › Figures S1 and S2, Tables S1 and S3.pdf]

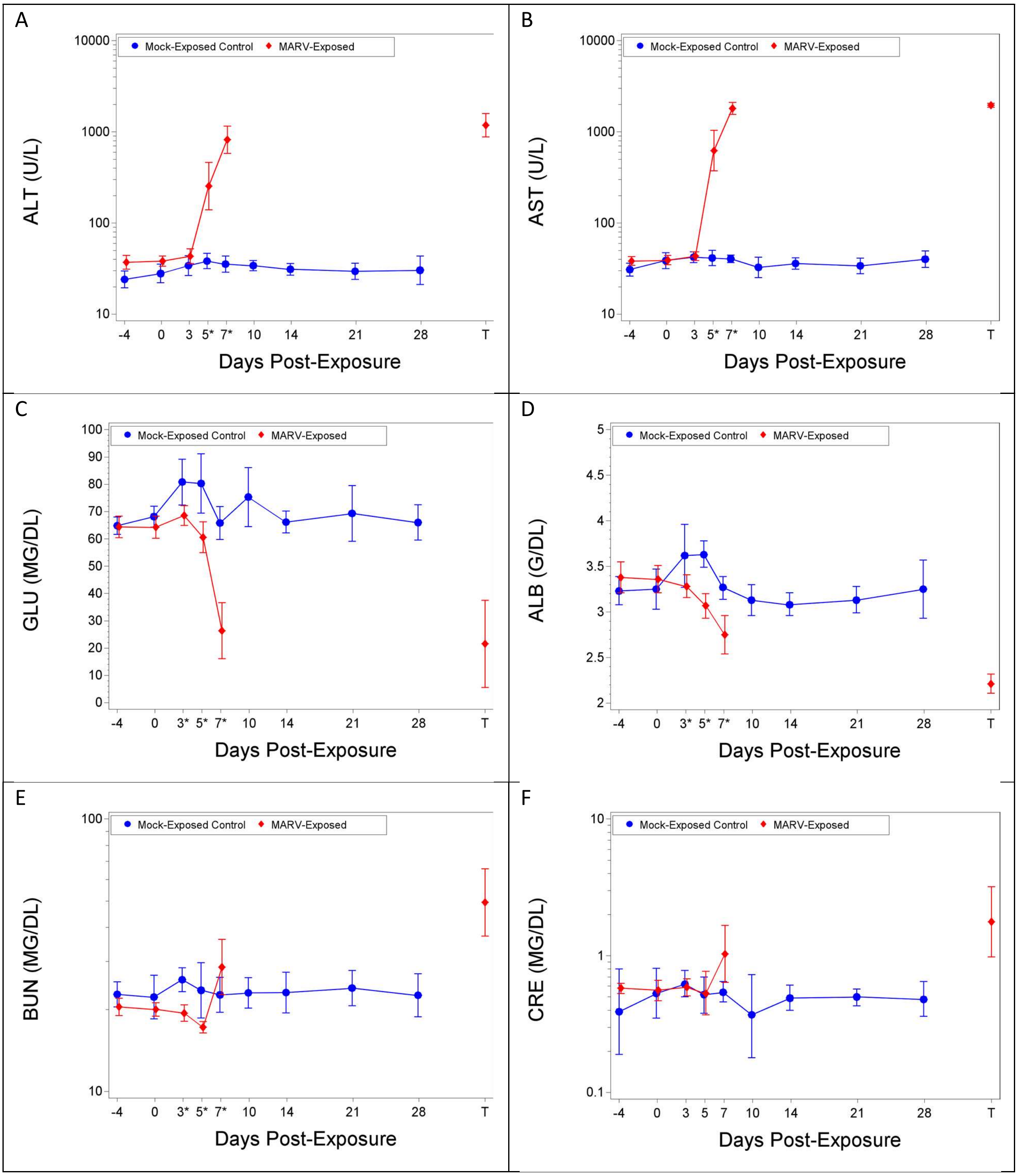
A
ALT (U/L)
Mock-Exposed Control
MARV-Exposed
10000
1000
100
10
-4 0 3 5* 7* 10 14 21 28 T
Days Post-Exposure
B
AST (U/L)
Mock-Exposed Control
MARV-Exposed
10000
1000
100
10
-4 0 3 5* 7* 10 14 21 28 T
Days Post-Exposure
C
GLU (MG/DL)
Mock-Exposed Control
MARV-Exposed
100 90 80 70 60 50 40 30 20 10 0
-4 0 3* 5* 7* 10 14 21 28 T
Days Post-Exposure
D
ALB (G/DL)
Mock-Exposed Control
MARV-Exposed
5 4.5 4 3.5 3 2.5 2
-4 0 3* 5* 7* 10 14 21 28 T
Days Post-Exposure
E
BUN (MG/DL)
Mock-Exposed Control
MARV-Exposed
100
10
-4 0 3* 5* 7* 10 14 21 28 T
Days Post-Exposure
F
CRE (MG/DL)
Mock-Exposed Control
MARV-Exposed
10
1
0.1
-4 0 3 5 7 10 14 21 28 T
Days Post-Exposure

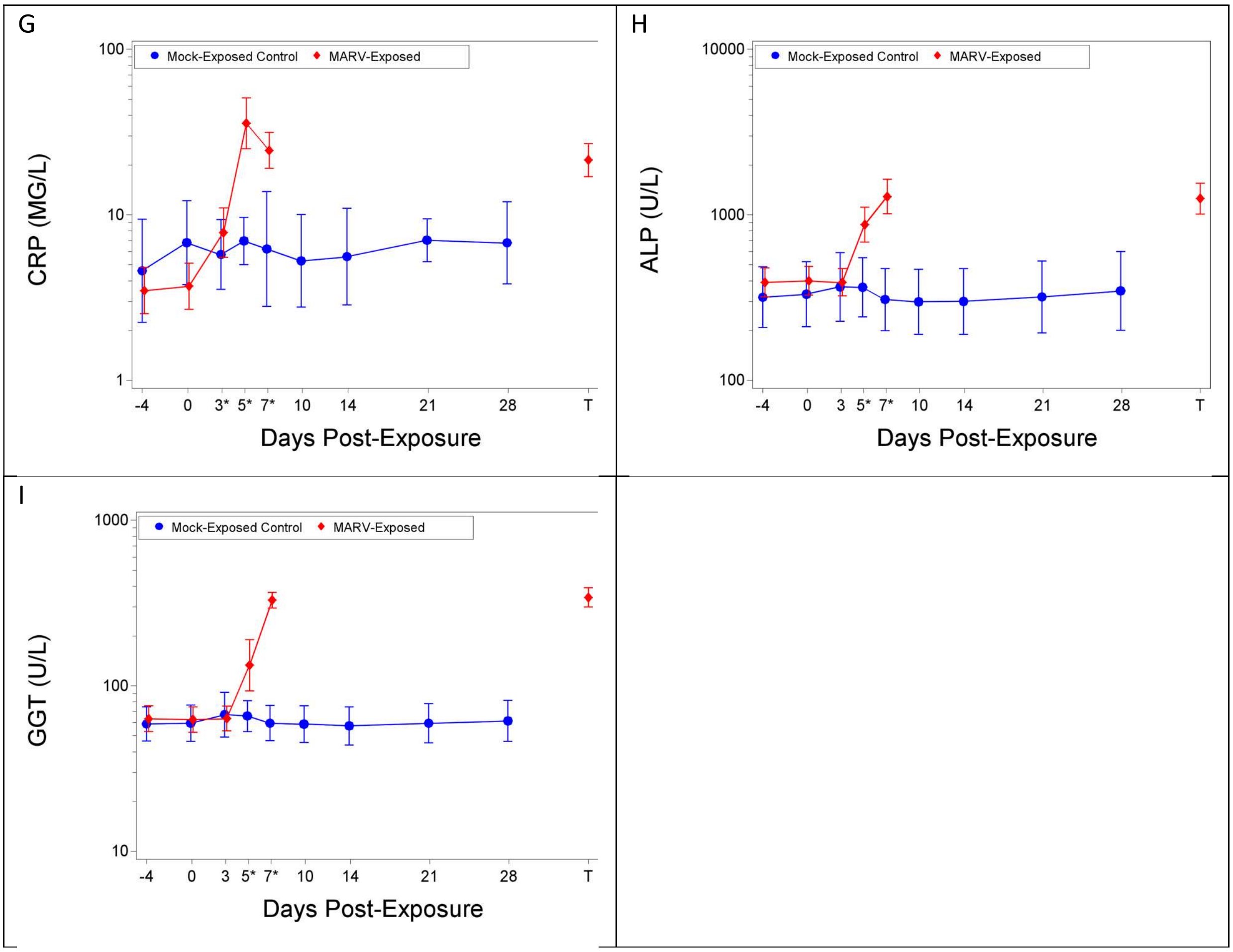

Figure S1. Serum Chemistry Changes over Time. The symbols represent geometric mean times and bars indicate 95% confidence intervals. * indicates statistically significant difference in means ($p<0.05$) between MARV-exposed and mock-exposed control groups.

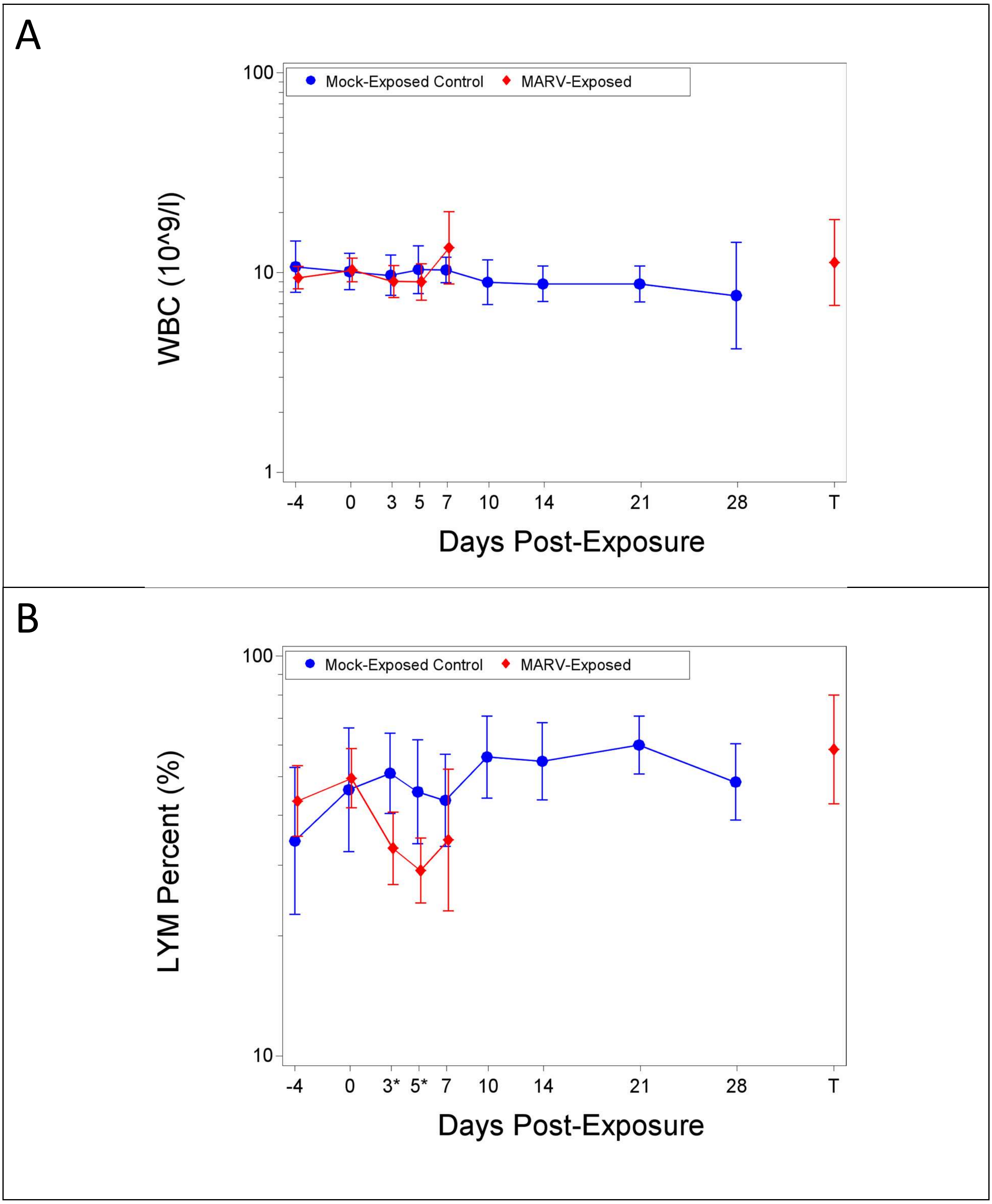
A
Mock-Exposed Control
MARV-Exposed
WBC (10^9/l)
100
10
1
-4 0 3 5 7 10 14 21 28 T
Days Post-Exposure
B
Mock-Exposed Control
MARV-Exposed
LYM Percent (%)
100
10
-4 0 3* 5* 7 10 14 21 28 T
Days Post-Exposure

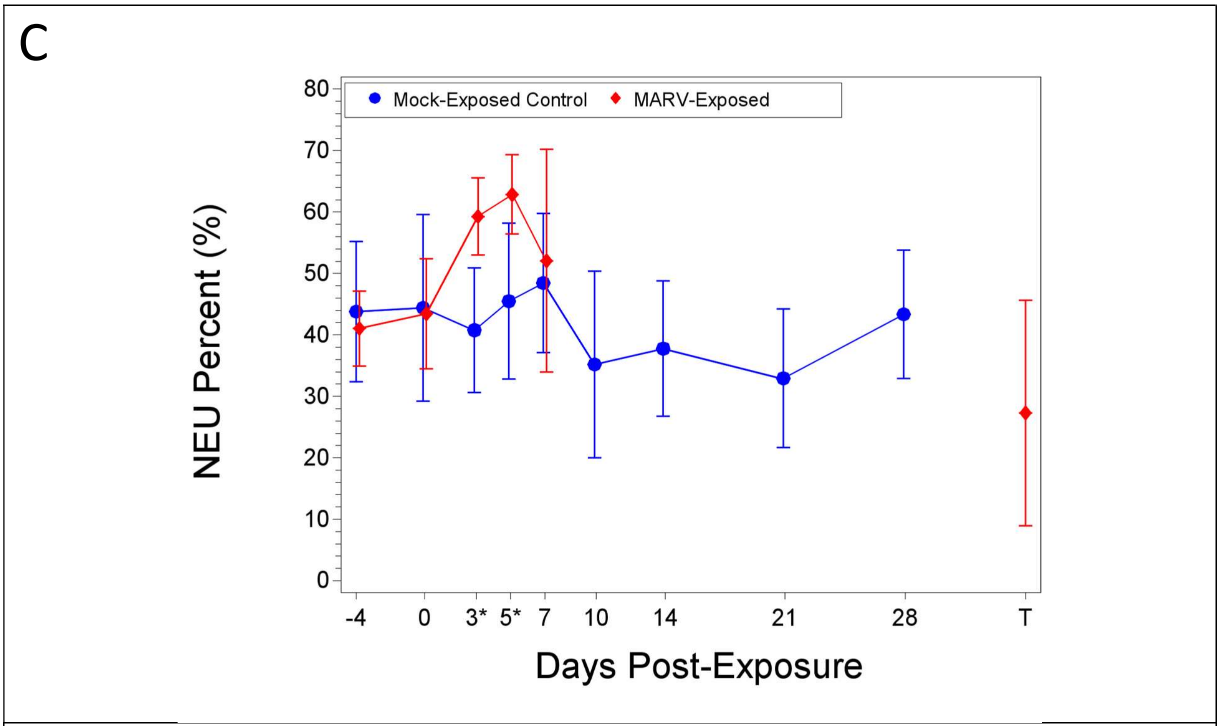

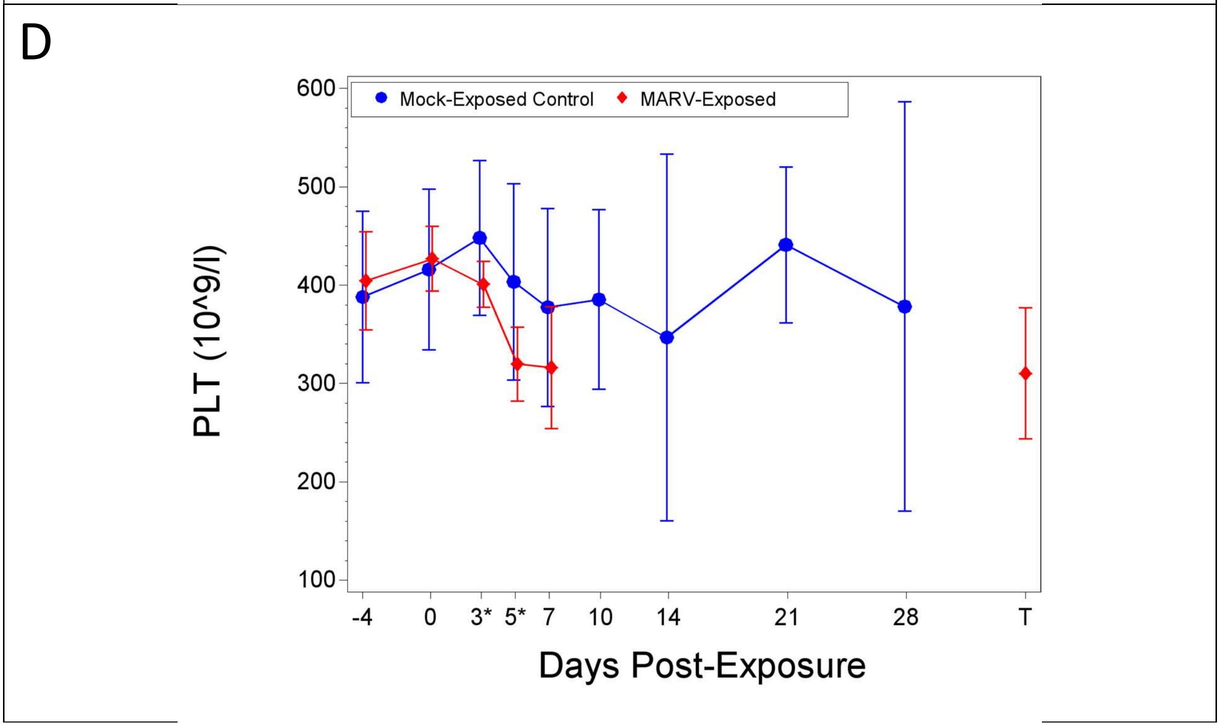

Figure S2. Hematological Changes over Time. The symbols represent geometric mean times and bars indicate 95% confidence intervals. * indicates statistically significant difference in means ($p<0.05$) between MARV-exposed and mock-exposed control groups.

Table S1. NHP Clinical Scoring Criteria.

Parameter	Degree of Parameter	Ob 1	Ob 2	Ob 3	Ob 4
	**Observation Start Time:**				
**Parameter**	**Degree of Parameter**	**Score (Circle One)**	**Score (Circle One)**	**Score (Circle One)**	**Score (Circle One)**
Respiration	Normal	0	0	0	0
	Abdominal breathing or labored breathing	4	4	4	4
	Severe dyspnea; agonal breathing	10	10	10	10
Food Consumption Feces/Urine	Normal	0	**Value for Observation 1 (Ob 1) should be carried over for Observations 2 through 4 (Ob 2, Ob 3, and Ob 4) as applicable.**		
	No biscuits eaten = 1<br>Consecutive days (Day 2=2, Day 3=3, Day 4=4, etc.)[1]				
	No enrichment eaten = 1<br>Consecutive days (Day 2=3, Day 3=4, Day 4=5, etc.)[2]				
	No feces seen (AM check); no urine seen (AM check)	1			
	Diarrhea (liquid)	2			
Activity/ Appearance	Normal	0	0	0	0
	Hunched but active most of the time	1	1	1	1
	Hunched with head between knees; dull appearance to eyes	3	3	3	3
	Lies down; gets up when approached	4	4	4	4
	Lies down; gets up with some prodding but not when approached	10	10	10	10
Bleeding/ Hemorrhage	No signs	0	0	0	0
	Petechiation and/or ecchymosis	2	2	2	2
	Observable bleeding; controlled by clotting (not menses)<br>**AND/OR** Petechiation and/or ecchymosis > 50%	4	4	4	4
	Uncontrolled bleeding	10	10	10	10
	**Total Score (sum of all circled values)**				
	**Observation End Time:**				
	Observations Performed by:	(Initial/Date)	(Initial/Date)	(Initial/Date)	(Initial/Date)
	Observations Recorded by:	(Initial/Date)	(Initial/Date)	(Initial/Date)	(Initial/Date)

Score = 0-3, no intervention. Score = ≥ 4 (or ≥ 3 in any single parameter), additional monitoring of at least once in the evening 4-6 hours after the final late afternoon check. Score ≥ 10, Euthanasia. [1] consecutive days with NO biscuit consumption only. [2] consecutive days with NO enrichment consumption only

Table S3. Historical Overview of Nonhuman Primate Controls Challenged with Marburg Virus: Time to Death.

Reference	NHP MARV infected	NHP Species	MARV Strain	Challenge Dose (PFU) and Route	Site	Mortality	Time to Death (Days)	Year Published
(1)<br>Ignatyev 1996	2	Rhesus	Popp	200 LD_{50} for guinea pigs, route not specified	Vector[1]	100%<br>(2/2)	10-11	1996
(2)<br>Hevey 1998	3	Cyno	MtE-Mus	8000 SC	USAMRIID[2]	100%<br>(3/3)	9-10	1998
(3)<br>Daddario-DiCaprio	1<br>1	Cyno	Angola<br>Ravn	1000<br>No route specified	USAMRIID	100%<br>(2/2)	8<br>8	2006
(4)<br>Daddario-DiCaprio	3	Rhesus	Musoke	1000 IM	USAMRIID	100%<br>(3/3)	11-12	2006
(5)<br>Geisbert 2007	6	Rhesus	Angola	1000 IM	USAMRIID	100%<br>(6/6)	7-8	2007
(6)<br>Geisbert 2008	2	Cyno	Musoke	1000 IN	USAMRIID	100%<br>(2/2)	10-13	2008
(7)<br>Swenson 2008	1<br>1<br>1	Cyno	Musoke<br>Ci67<br>Ravn	1000 SC	USAMRIID	100%<br>(1/1)<br>100%<br>(1/1)<br>100%<br>(1/1)	10<br>10<br>8	2008
(8)<br>Alves 2010	6<br>(4M/2F)	Cyno	Angola	IN: 2<br>11<br>14<br>339<br>99<br>705	USAMRIID	100%<br>(6/6)	9<br>9<br>9<br>8<br>8<br>7	2010
(9)<br>Geisbert 2010a	3	Cyno	Angola	1000 IM	USAMRIID	100%<br>(3/3)	8-9	2010
(10)<br>Geisbert 2010b	3	Rhesus	Musoke	1000 IM	USAMRIID	100%<br>(3/3)	11-12	2010
(11)<br>Hensley	18	Cyno	Ci67	10000 IM	USAMRIID	Serial sac	NA	2011
(12)<br>Dye 2012	4	Rhesus	Not specified	1000 IM	USAMRIID	100%<br>(4/4)	8-10	2012
(13)<br>Smith 2013	6	Rhesus	Musoke	1000 IM	USAMRIID	100%<br>(6/6)	10-12	2013
(14)<br>Mire 2014	2	Cyno	Musoke	1000 IM	UTMB	100%<br>(2/2)	10-11	2014

[1] State Scientific Centre of Virology and Biotechnology, Koltsovo, Novosibirsk Region, Russia
[2] US Army Medical Research Institute of Infectious Diseases

(15)<br>Thi 2014	5 (3M/2F)	Rhesus	Angola	1000 IM	UTMB	100%<br>(5/5)	7-9	2014
(16)<br>Johnston 2015	8<br>8<br>8<br>8<br>8<br>10<br>10	Cyno	Angola	Target/Actual<br>1/0 IM<br>10/18 IM<br>100/260 IM<br>1000/2520 IM<br>10,000/29,200 IM<br>10/33 IN<br>100/235 IN	USAMRIID	100%<br>(60/60)	11<br>10<br>10<br>9<br>9<br>10<br>11	2015
(17)<br>Fernando 2015	3<br>(3F)	Cyno	Angola	1000 $TCID_{50}$ IM	Health Canada	100%<br>(3/3)	6-7	2015
(18)<br>Heald 2015	30<br>(20M/10F)	Cyno	Musoke	1000 SC	USAMRIID	100%<br>(30/30)	13	2015
(19)<br>Dye 2016	3	Cyno	Musoke	Target/Actual<br>1000/315 SC<br>1000/113 IN	USAMRIID	100%<br>(3/3)	10	2016
(20)<br>Warren 2016	6 (4M/2F)	Cyno	Musoke	1830 SC	USAMRIID	100%<br>(6/6)	9-11	2016
(21)<br>Ewers 2016	5<br>(3M/2F)	Rhesus	Angola	675-7630 IN	USAMRIID	100%<br>(5/5)	7-8	2016
(22)<br>Mire 2017	2<br>1	Rhesus	Angola<br>Ravn	Target/Actual<br>1000/1050-1240<br>1000/1100 IM	UTMB	100%<br>(2/2)<br>(1/1)	8-9<br>10	2017
(23)<br>Thi 2017	2<br>2	Rhesus	Angola<br>Ravn	1000 IM	UTBM	100%<br>(2/2)	8<br>7, 10	2017
(24)<br>Callendret 2018	2	Cyno	Angola	1000 IM	TBRI[3] &<br>UTMB[4]	100%<br>(2/2)	10	2018
(25)<br>Matassov 2018	2	Cyno	Angola	1000 IM	UTMB	100%<br>(2/2)	7-9	2018
(26)<br>Cooper 2018	6<br>(2M/4F)	Rhesus	Angola	1000 IM	NIAID IRF[5]	100%<br>(6/6)	9	2018

[3] Texas Biomedical Research Institute
[4] University of Texas Medical Branch
[5] National Institutes of Allergy and Infectious Disease Integrated Research Facility
Cyno, cynomolgus macaque
Rhesus, rhesus macaque
IN, intranasal challenge
IM, intramuscular
SC, subcutaneous

(27)<br>Nicholas 2018	2<br>2<br>2<br>1<br>2<br>2<br>2<br>1	Rhesus<br>“<br>“<br>“<br>Cyno<br>“<br>“<br>“	Angola<br>Musoke<br>Ravn<br>Ozolin<br>Angola<br>Musoke<br>Ravn<br>Ozolin	1000 IM	NIAID	100% (2/2)<br>100% (2.2)<br>0% (0/2)<br>0% (0/1)<br>100% (2/2)<br>50% (1/2)<br>100% (2/2)<br>0% (0/1)	7-8<br>9-16<br>>22<br>>22<br>7<br>9<br>8, >22<br>>22	2018
(28)<br>Woolsey 2018	1<br>4	Rhesus	Angola	1000 IM<br>50 IM	UTMB	100% (5/5)	8<br>8-12	2018
(29)<br>Blair 2018	47	Cyno	18 Angola<br>29 Musoke	1000 IM	USAMRIID	100% for Angola	8.13	2018
(30)<br>Porter 2020	6	Cyno	Angola	1000 IM	USAMRIID	100% (6/6)	7-9	2020
(31)<br>Woolsey 2022	3	Cyno	Angola	1000 IM	UTMB	100% (3/3)	8-9	2022

1. Ignatyev GM, Agafonov AP, Streltsova MA, Kashentseva EA. Inactivated Marburg virus elicits a nonprotective immune response in Rhesus monkeys. Journal of Biotechnology. 1996;44:111-8.
2. Hevey M, Negley D, Pushko P, Smith J, Schmaljoh A. Marburg virus vaccines based upon Alphavirus replicons protect guinea pigs and nonhuman primates. Virology. 1998;251:28-37.
3. Daddario-DiCaprio KM, Geisbert TW, Geisbert JB, Stroher U, Hensley LE, Grolla A, et al. Cross-protection against Marburg virus strains by using a live, attenuated recombinant vaccine. J Virol. 2006;80(19):9659-66.
4. Daddario-DiCaprio KM, Geisbert TW, Ströher U, Geisbert JB, Grolla A, Fritz EA, et al. Postexposure protection against Marburg haemorrhagic fever with recombinant vesicular stomatitis virus vectors in non-human primates: an efficacy assessment. The Lancet. 2006;367(9520):1399-404.
5. Geisbert TW, Daddario-DiCaprio KM, Geisbert JB, Young HA, Formenty P, Fritz EA, et al. Marburg virus Angola infection of rhesus macaques: pathogenesis and treatment with recombinant nematode anticoagulant protein c2. J Infect Dis. 2007;196 Suppl 2:S372-81.
6. Geisbert TW, Daddario-Dicaprio KM, Geisbert JB, Reed DS, Feldmann F, Grolla A, et al. Vesicular stomatitis virus-based vaccines protect nonhuman primates against aerosol challenge with Ebola and Marburg viruses. Vaccine. 2008;26(52):6894-900.
7. Swenson DL, Warfield KL, Larsen T, Alves DA, Coberley SS, Bavari S. Monovalent virus-like particle vaccine protects guinea pigs and nonhuman primates against infection with multiple Marburg viruses. Expert Reviews Vaccines. 2008;7(4):417-29.
8. Alves DA, Glynn AR, Steele KE, Lackemeyer MG, Garza NL, Buck JG, et al. Aerosol exposure to the angola strain of marburg virus causes lethal viral hemorrhagic Fever in cynomolgus macaques. Vet Pathol. 2010;47(5):831-51.
9. Geisbert TW, Bailey M, Geisbert JB, Asiedu C, Roederer M, Grazia-Pau M, et al. Vector choice determines immunogenicity and potency of genetic vaccines against Angola Marburg virus in nonhuman primates. J Virol. 2010;84(19):10386-94.
10. Geisbert TW, Hensley LE, Geisbert JB, Leung A, Johnson JC, Grolla A, et al. Postexposure treatment of Marburg virus infection. Emerg Infect Dis. 2010;16(7):1119-22.
11. Lisa E. Hensley, Derron A. Alves, Joan B. Geisbert, Elizabeth A. Fritz, Christopher Reed, Tom Larsen, Thomas W. Geisbert. Pathogenesis of Marburg Hemorrhagic Fever in Cynomolgus macaques. JID 2011;204 (Suppl 3), S1021-1031.
12. Dye JM, Herbert AS, Kuehne AI, Barth JF, Muhammad MA, Zak SE, et al. Postexposure antibody prophylaxis protects nonhuman primates from filovirus disease. Proc Natl Acad Sci U S A. 2012;109(13):5034-9.
13. Smith LM, Hensley LE, Geisbert TW, Johnson J, Stossel A, Honko A, et al. Interferon-beta therapy prolongs survival in rhesus macaque models of Ebola and Marburg hemorrhagic fever. J Infect Dis. 2013;208(2):310-8.
14. Mire CE, Geisbert JB, Agans KN, Satterfield BA, Versteeg KM, Fritz EA, et al. Durability of a vesicular stomatitis virus-based marburg virus vaccine in nonhuman primates. PLoS One. 2014;9(4):e94355.
15. Thi EP, Mire CE, Ursic-Bedoya R, Geisbert JB, Lee ACH, Agans KN, et al. Marburg virus infection in nonhuman primates: Therapeutic treatment by lipid-encapsulated siRNA. Science Translational Medicine. 2014;6(250):1-11.
16. Johnston SC, Lin KL, Twenhafel NA, Raymond JL, Shamblin JD, Wollen SE, et al. Dose Response of MARV/Angola Infection in cynomolgus macaques following IM or aerosol exposure. PLoS One. 2015;10(9):e0138843.
17. Fernando L, Qiu X, Melito PL, Williams KJ, Feldmann F, Feldmann H, et al. Immune response to Marburg Virus Angola infection in nonhuman primates. J Infect Dis. 2015;212 Suppl 2:S234-41.

18. Heald AE, Charleston JS, Iversen PL, Warren TK, Saoud JB, Al-Ibrahim M, et al. AVI-7288 for Marburg Virus in nonhuman primates and humans. N Engl J Med. 2015;373(4):339-48.
19. Dye JM, Warfield KL, Wells JB, Unfer RC, Shulenin S, Vu H, et al. Virus-Like Particle vaccination protects nonhuman primates from lethal aerosol exposure with Marburgvirus (VLP Vaccination Protects Macaques against Aerosol Challenges). Viruses. 2016;8(4):94.
20. Warren TK, Whitehouse CA, Wells J, Welch L, Charleston JS, Heald A, et al. Delayed time-to-treatment of an antisense morpholino oligomer is effective against lethal Marburg Virus infection in cynomolgus macaques. PLoS Negl Trop Dis. 2016;10(2):e0004456.
21. Ewers EC, Pratt WD, Twenhafel NA, Shamblin J, Donnelly G, Esham H, et al. Natural history of aerosol exposure with Marburg virus in Rhesus macaques. Viruses. 2016;8(4):87.
22. Mire CE, Geisbert JB, Borisevich V, Fenton KA, Agans KN, Flyak AI, et al. Therapeutic treatment of Marburg and Ravn virus infection in nonhuman primates with a human monoclonal antibody. Science Translational Medicine. 2017;9:1-9.
23. Thi EP, Mire CE, Lee AC, Geisbert JB, Ursic-Bedoya R, Agans KN, et al. siRNA rescues nonhuman primates from advanced Marburg and Ravn virus disease. J Clin Invest. 2017;127(12):4437-48.
24. Callendret B, Vellinga J, Wunderlich K, Rodriguez A, Steigerwald R, Dirmeier U, et al. A prophylactic multivalent vaccine against different filovirus species is immunogenic and provides protection from lethal infections with Ebolavirus and Marburgvirus species in non-human primates. PLoS One. 2018;13(2):e0192312.
25. Matassov D, Mire CE, Latham T, Geisbert JB, Xu R, Ota-Setlik A, et al. Single-dose trivalent VesiculoVax vaccine protects macaques from lethal Ebolavirus and Marburgvirus challenge. Journal of Virology. 2018;92(3):1-20.
26. Cooper TK, Sword J, Johnson JC, Bonilla A, Hart R, Liu DX, et al. New insights into Marburg virus disease pathogenesis in the Rhesus macaque model. J Infect Dis. 2018;218(suppl_5):S423-S33.
27. Nicholas VV, Rosenke R, Feldmann F, Long D, Thomas T, Scott DP, et al. Distinct biological phenotypes of Marburg and Ravn virus infection in macaques. J Infect Dis. 2018;218(suppl_5):S458-S65.
28. Woolsey C, Geisbert JB, Matassov D, Agans KN, Borisevich V, Cross RW, et al. Postexposure efficacy of recombinant Vesicular Stomatitis Virus vectors against high and low doses of Marburg virus variant Angola in nonhuman primates. J Infect Dis. 2018;218(suppl_5):S582-S7.
29. Blair PW, Keshtkar-Jahromi M, Psoter KJ, Reisler RB, Warren TK, Johnston S, Goff AJ, Downey LG, Bavari S, Cardile A. Virulence of Marburg virus Angola compared to Mt. Elgon (Musoke) in macaques: A Pooled Survival Analysis. Viruses 2018;10, 658; doi:10.3390/v10110658.
30. Porter DP, Weidner JM, Gomba L, Bannister R, Blair C, Jordan R, et al. Remdesivir (GS-5734) is efficacious in cynomolgus macaques infected with Marburg virus. J Infect Dis. 2020;222, 1894-1901.
31. Woolsey C, Cross RW, Agans KN, Borisevich V, Deer DJ, Geisbert JB, et al. A highly attenuated Vesiculovax vaccine rapidly protects nonhuman primates against lethal Marburg virus challenge. PLoS Negl Trop Dis 2022;16(5): e0010433. https://doi.org/10.1371/journal. pntd.0010433.
