# Supplementary material for: Natural History of Marburg Virus Infection to Support Medical Countermeasure Development
Source: Viruses. 2022 Oct 18;14(10):2291. doi: 10.3390/v14102291 (PMC9607268; doi:10.3390/v14102291)
Supplement: Supplementary file 1 [file viruses-14-02291-s001.zip › Table S4 MARV Exposed Clinical Scores.pdf]

Table S4. Clinical Scoring for MARV-exposed NHPs

Clinical Scoring: MARV-exposed NHP 1

Parameter	Degree of Parameter	Score[a]	Score by Study Day																								
			-4		-3		-2		-1		0		1		2		3		4		5		6		7		
			Ob 1	Ob 2	Ob 1	Ob 2	Ob 1	Ob 2	Ob 1	Ob 2	Ob 1	Ob 2	Ob 1	Ob 2	Ob 1	Ob 2	Ob 1	Ob 2	Ob 1	Ob 2	Ob 1	Ob 2	Ob 1	Ob 2	Ob 1	Ob 2	Ob 3
Respiration	Normal	0	0	0	0	0	0	0	0	0	0	0	0	0	0	0	0	0	0	0	0	0	0	0	0	0	
	Abdominal breathing or labored breathing	4																									4
	Severe dyspnea; agonal breathing	10																									
Food Consumption Feces/Urine	Normal	0	0		0		0		0		0		0		0		0		0		0		0				
	No biscuits eaten	1																							1	1	1
	Consecutive days (Day 2=2, Day 3=3, Day 4=4, etc.)[1]	A																									
	No enrichment eaten	1																									
	Consecutive days (Day 2=3, Day 3=4, Day 4=5, etc.)[2]	A																									
	No feces seen (AM check); no urine seen (AM check)	1																									
	Diarrhea (liquid)	2																									
Activity/ Appearance	Normal	0	0	0	0	0	0	0	0	0	0	0	0	0	0	0	0	0	0	0	0	0	0	0	0		
	Hunched but active most of the time	1																								1	1
	Hunched with head between knees; dull appearance to eyes	3																									
	Lies down; gets up when approached	4																									
	Lies down; gets up with some prodding but not when approached	10																									
Bleeding/ Hemorrhage	No Signs	0	0	0	0	0	0	0	0	0	0	0	0	0	0	0	0	0	0	0	0	0	0	0			
	Petechiation and/or ecchymosis	2																									
	Observable bleeding; controlled by clotting (not menses) AND/OR Petechiation and/or ecchymosis > 50%	4																							4	4	4
	Uncontrolled Bleeding	10																									
		**Total Score**	**0**	**0**	**0**	**0**	**0**	**0**	**0**	**0**	**0**	**0**	**0**	**0**	**0**	**0**	**0**	**0**	**0**	**0**	**0**	**0**	**0**	**0**	**5**	**6**	**10**

[a] Score = 0-3, no intervention. Score = ≥ 4 (or ≥ 3 in any single parameter), additional monitoring of at least once in the evening 4-6 hours after the final late afternoon check. Score

[1] Consecutive days with NO biscuit consumption only.

[2] Consecutive days with NO enrichment consumption only.

A=As applicable

Clinical Scoring: MARV-exposed NHP 2																										
Parameter	Degree of Parameter	Score[a]	Score by Study Day																							
			-4		-3		-2		-1		0		1		2		3		4		5		6			7
			Ob 1	Ob 2	Ob 1	Ob 2	Ob 1	Ob 2	Ob 1	Ob 2	Ob 1	Ob 2	Ob 1	Ob 2	Ob 1	Ob 2	Ob 1	Ob 2	Ob 1	Ob 2	Ob 1	Ob 2	Ob 1	Ob 2	Ob 3	Ob 1
Respiration	Normal	0	0	0	0	0	0	0	0	0	0	0	0	0	0	0	0	0	0	0	0	0	0	0	0	NS
	Abdominal breathing or labored breathing	4																								
	Severe dyspnea; agonal breathing	10																								
Food Consumption Feces/Urine	Normal	0	0		0		0		0		0		0		0		0		0		0					
	No biscuits eaten	1																					1	1	1	
	Consecutive days (Day 2=2, Day 3=3, Day 4=4, etc.)[1]	A																								
	No enrichment eaten	1																								
	Consecutive days (Day 2=3, Day 3=4, Day 4=5, etc.)[2]	A																								
	No feces seen (AM check); no urine seen (AM check)	1																								
	Diarrhea (liquid)	2																								
Activity/ Appearance	Normal	0	0	0	0	0	0	0	0	0	0	0	0	0	0	0	0	0	0	0	0	0				NS
	Hunched but active most of the time	1																					1	1	1	
	Hunched with head between knees; dull appearance to eyes	3																								
	Lies down; gets up when approached	4																								
	Lies down; gets up with some prodding but not when approached	10																								
Bleeding/ Hemorrhage	No Signs	0	0	0	0	0	0	0	0	0	0	0	0	0	0	0	0	0	0	0						NS
	Petechiation and/or ecchymosis	2																			2	2	2			
	Observable bleeding; controlled by clotting (not menses) AND/OR Petechiation and/or ecchymosis > 50%	4																						4	4	
	Uncontrolled Bleeding	10																								
		**Total Score**	**0**	**0**	**0**	**0**	**0**	**0**	**0**	**0**	**0**	**0**	**0**	**0**	**0**	**0**	**0**	**0**	**0**	**0**	**2**	**2**	**4**	**6**	**6**	**FD**

[a] Score = 0-3, no intervention. Score = ≥ 4 (or ≥ 3 in any single parameter), additional monitoring of at least once in the evening 4-6 hours after the final late afternoon check. Score

[1] Consecutive days with NO biscuit consumption only.

[2] Consecutive days with NO enrichment consumption only.

A=As applicable

Clinical Scoring: MARV-exposed NHP 3																											
Parameter	Degree of Parameter	Score[a]	Score by Study Day																								
			-4		-3		-2		-1		0		1		2		3		4		5		6			7	
			Ob 1	Ob 2	Ob 1	Ob 2	Ob 1	Ob 2	Ob 1	Ob 2	Ob 1	Ob 2	Ob 1	Ob 2	Ob 1	Ob 2	Ob 1	Ob 2	Ob 1	Ob 2	Ob 1	Ob 2	Ob 1	Ob 2	Ob 3	Ob 1	Ob 2
Respiration	Normal	0	0	0	0	0	0	0	0	0	0	0	0	0	0	0	0	0	0	0	0	0	0	0	0	0	
	Abdominal breathing or labored breathing	4																									4
	Severe dyspnea; agonal breathing	10																									
Food Consumption Feces/Urine	Normal	0	0		0		0		0		0		0		0		0		0		0						
	No biscuits eaten	1																					1	1	1		
	Consecutive days (Day 2=2, Day 3=3, Day 4=4, etc.)[1]	A																								2	2
	No enrichment eaten	1																									
	Consecutive days (Day 2=3, Day 3=4, Day 4=5, etc.)[2]	A																									
	No feces seen (AM check); no urine seen (AM check)	1																									
	Diarrhea (liquid)	2																									
Activity/ Appearance	Normal	0	0	0	0	0	0	0	0	0	0	0	0	0	0	0	0	0	0	0	0	0					
	Hunched but active most of the time	1																					1	1			
	Hunched with head between knees; dull appearance to eyes	3																							3	3	
	Lies down; gets up when approached	4																									
	Lies down; gets up with some prodding but not when approached	10																									10
Bleeding/ Hemorrhage	No Signs	0	0	0	0	0	0	0	0	0	0	0	0	0	0	0	0	0	0	0							
	Petechiation and/or ecchymosis	2																			2	2	2	2	2		
	Observable bleeding; controlled by clotting (not menses) AND/OR Petechiation and/or ecchymosis > 50%	4																								4	4
	Uncontrolled Bleeding	10																									
		**Total Score**	**0**	**0**	**0**	**0**	**0**	**0**	**0**	**0**	**0**	**0**	**0**	**0**	**0**	**0**	**0**	**0**	**0**	**0**	**2**	**2**	**4**	**4**	**6**	**9**	**20**

[a] Score = 0-3, no intervention. Score = ≥ 4 (or ≥ 3 in any single parameter), additional monitoring of at least once in the evening 4-6 hours after the final late afternoon check. Score

[1] Consecutive days with NO biscuit consumption only.

[2] Consecutive days with NO enrichment consumption only.

A=As applicable

Clinical Scoring: MARV-exposed NHP 4																													
Parameter	Degree of Parameter	Score[a]	Score by Study Day																									
			-4		-3		-2		-1		0		1		2		3		4		5		6			7		
			Ob 1	Ob 2	Ob 1	Ob 2	Ob 1	Ob 2	Ob 1	Ob 2	Ob 1	Ob 2	Ob 1	Ob 2	Ob 1	Ob 2	Ob 1	Ob 2	Ob 1	Ob 2	Ob 1	Ob 2	Ob 1	Ob 2	Ob 3	Ob 1	Ob 2	Ob 3
Respiration	Normal	0	0	0	0	0	0	0	0	0	0	0	0	0	0	0	0	0	0	0	0	0	0	0	0	0	0	
	Abdominal breathing or labored breathing	4																										4
	Severe dyspnea; agonal breathing	10																										
Food Consumption Feces/Urine	Normal	0	0		0		0		0		0		0		0		0		0		0							
	No biscuits eaten	1																					1	1	1			
	Consecutive days (Day 2=2, Day 3=3, Day 4=4, etc.)[1]	A																								2	2	2
	No enrichment eaten	1																										
	Consecutive days (Day 2=3, Day 3=4, Day 4=5, etc.)[2]	A																										
	No feces seen (AM check); no urine seen (AM check)	1																										
	Diarrhea (liquid)	2																										
Activity/ Appearance	Normal	0	0	0	0	0	0	0	0	0	0	0	0	0	0	0	0	0	0	0	0	0	0	0				
	Hunched but active most of the time	1																							1	1	1	
	Hunched with head between knees; dull appearance to eyes	3																										3
	Lies down; gets up when approached	4																										
	Lies down; gets up with some prodding but not when approached	10																										
Bleeding/ Hemorrhage	No Signs	0	0	0	0	0	0	0	0	0	0	0	0	0	0	0	0	0	0	0	0	0	0	0	0			
	Petechiation and/or ecchymosis	2																								2	2	2
	Observable bleeding; controlled by clotting (not menses) AND/OR Petechiation and/or ecchymosis > 50%	4																										
	Uncontrolled Bleeding	10																										
**Total Score**			**0**	**0**	**0**	**0**	**0**	**0**	**0**	**0**	**0**	**0**	**0**	**0**	**0**	**0**	**0**	**0**	**0**	**0**	**0**	**0**	**1**	**1**	**2**	**5**	**5**	**11**

[a] Score = 0-3, no intervention. Score = ≥ 4 (or ≥ 3 in any single parameter), additional monitoring of at least once in the evening 4-6 hours after the final late afternoon check. Score

[1] Consecutive days with NO biscuit consumption only.

[2] Consecutive days with NO enrichment consumption only.

A=As applicable

Clinical Scoring: MARV-exposed NHP 5																												
Parameter	Degree of Parameter	Score[a]	Score by Study Day																									
			-4		-3		-2		-1		0		1		2		3		4		5		6			7		
			Ob 1	Ob 2	Ob 1	Ob 2	Ob 1	Ob 2	Ob 1	Ob 2	Ob 1	Ob 2	Ob 1	Ob 2	Ob 1	Ob 2	Ob 1	Ob 2	Ob 1	Ob 2	Ob 1	Ob 2	Ob 1	Ob 2	Ob 3	Ob 1	Ob 2	Ob 3
Respiration	Normal	0	0	0	0	0	0	0	0	0	0	0	0	0	0	0	0	0	0	0	0	0	0	0	0	0	0	
	Abdominal breathing or labored breathing	4																										4
	Severe dyspnea; agonal breathing	10																										
Food Consumption Feces/Urine	Normal	0	0		0		0		0		0		0		0		0		0		0							
	No biscuits eaten	1																					1	1	1			
	Consecutive days (Day 2=2, Day 3=3, Day 4=4, etc.)[1]	A																								2	2	2
	No enrichment eaten	1																										
	Consecutive days (Day 2=3, Day 3=4, Day 4=5, etc.)[2]	A																										
	No feces seen (AM check); no urine seen (AM check)	1																										
	Diarrhea (liquid)	2																										
Activity/ Appearance	Normal	0	0	0	0	0	0	0	0	0	0	0	0	0	0	0	0	0	0	0	0	0	0	0	0			
	Hunched but active most of the time	1																								1	1	
	Hunched with head between knees; dull appearance to eyes	3																										3
	Lies down; gets up when approached	4																										
	Lies down; gets up with some prodding but not when approached	10																										
Bleeding/ Hemorrhage	No Signs	0	0	0	0	0	0	0	0	0	0	0	0	0	0	0	0	0	0	0	0	0	0	0	0			
	Petechiation and/or ecchymosis	2																										
	Observable bleeding; controlled by clotting (not menses) AND/OR Petechiation and/or ecchymosis > 50%	4																								4	4	4
	Uncontrolled Bleeding	10																										
		**Total Score**	**0**	**0**	**0**	**0**	**0**	**0**	**0**	**0**	**0**	**0**	**0**	**0**	**0**	**0**	**0**	**0**	**0**	**0**	**0**	**0**	**1**	**1**	**1**	**7**	**7**	**13**

[a] Score = 0-3, no intervention. Score = ≥ 4 (or ≥ 3 in any single parameter), additional monitoring of at least once in the evening 4-6 hours after the final late afternoon check. Score

[1] Consecutive days with NO biscuit consumption only.

[2] Consecutive days with NO enrichment consumption only.

A=As applicable

Clinical Scoring: MARV-exposed NHP 6

Parameter	Degree of Parameter	Score[a]	Score by Study Day																											
			-4		-3		-2		-1		0		1		2		3		4		5		6			7			8	
			Ob 1	Ob 2	Ob 1	Ob 2	Ob 1	Ob 2	Ob 1	Ob 2	Ob 1	Ob 2	Ob 1	Ob 2	Ob 1	Ob 2	Ob 1	Ob 2	Ob 1	Ob 2	Ob 1	Ob 2	Ob 1	Ob 2	Ob 3	Ob 1	Ob 2	Ob 3	Ob 1	Ob 2
Respiration	Normal	0	0	0	0	0	0	0	0	0	0	0	0	0	0	0	0	0	0	0	0	0	0	0	0	0	0	0	0	0
	Abdominal breathing or labored breathing	4																												
	Severe dyspnea; agonal breathing	10																												
Food Consumption Feces/Urine	Normal	0	0		0		0		0		0		0		0		0		0		0									
	No biscuits eaten	1																					1	1	1					
	Consecutive days (Day 2=2, Day 3=3, Day 4=4, etc.)[1]	A																								2	2	2	3	3
	No enrichment eaten	1																												
	Consecutive days (Day 2=3, Day 3=4, Day 4=5, etc.)[2]	A																												
	No feces seen (AM check); no urine seen (AM check)	1																												
	Diarrhea (liquid)	2																												
Activity/ Appearance	Normal	0	0	0	0	0	0	0	0	0	0	0	0	0	0	0	0	0	0	0	0	0	0	0	0	0				
	Hunched but active most of the time	1																									1	1		
	Hunched with head between knees; dull appearance to eyes	3																											3	
	Lies down; gets up when approached	4																												
	Lies down; gets up with some prodding but not when approached	10																												10
Bleeding/ Hemorrhage	No Signs	0	0	0	0	0	0	0	0	0	0	0	0	0	0	0	0	0	0	0	0	0	0	0	0					
	Petechiation and/or ecchymosis	2																								2	2	2	2	2
	Observable bleeding; controlled by clotting (not menses) AND/OR Petechiation and/or ecchymosis > 50%	4																												
	Uncontrolled Bleeding	10																												
		**Total Score**	**0**	**0**	**0**	**0**	**0**	**0**	**0**	**0**	**0**	**0**	**0**	**0**	**0**	**0**	**0**	**0**	**0**	**0**	**0**	**0**	**1**	**1**	**1**	**4**	**5**	**5**	**8**	**15**

[a] Score = 0-3, no intervention. Score = ≥ 4 (or ≥ 3 in any single parameter), additional monitoring of at least once in the evening 4-6 hours after the final late afternoon check. Score

[1] Consecutive days with NO biscuit consumption only.

[2] Consecutive days with NO enrichment consumption only.

A=As applicable

Clinical Scoring: MARV-exposed NHP 7																									
Parameter	Degree of Parameter	Score[a]	Score by Study Day																						
			-4		-3		-2		-1		0		1		2		3		4		5		6		
			Ob 1	Ob 2	Ob 1	Ob 2	Ob 1	Ob 2	Ob 1	Ob 2	Ob 1	Ob 2	Ob 1	Ob 2	Ob 1	Ob 2	Ob 1	Ob 2	Ob 1	Ob 2	Ob 1	Ob 2	Ob 1	Ob 2	Ob 3
Respiration	Normal	0	0	0	0	0	0	0	0	0	0	0	0	0	0	0	0	0	0	0	0	0	0	0	
	Abdominal breathing or labored breathing	4																							
	Severe dyspnea; agonal breathing	10																							10
Food Consumption Feces/Urine	Normal	0	0		0		0		0		0		0		0		0		0		0				
	No biscuits eaten	1																					1	1	1
	Consecutive days (Day 2=2, Day 3=3, Day 4=4, etc.)[1]	A																							
	No enrichment eaten	1																							
	Consecutive days (Day 2=3, Day 3=4, Day 4=5, etc.)[2]	A																							
	No feces seen (AM check); no urine seen (AM check)	1																							
	Diarrhea (liquid)	2																							
Activity/ Appearance	Normal	0	0	0	0	0	0	0	0	0	0	0	0	0	0	0	0	0	0	0	0	0			
	Hunched but active most of the time	1																					1		
	Hunched with head between knees; dull appearance to eyes	3																						3	
	Lies down; gets up when approached	4																							
	Lies down; gets up with some prodding but not when approached	10																							10
Bleeding/ Hemorrhage	No Signs	0	0	0	0	0	0	0	0	0	0	0	0	0	0	0	0	0	0	0					
	Petechiation and/or ecchymosis	2																			2	2	2	2	2
	Observable bleeding; controlled by clotting (not menses) AND/OR Petechiation and/or ecchymosis > 50%	4																							
	Uncontrolled Bleeding	10																							
		**Total Score**	**0**	**0**	**0**	**0**	**0**	**0**	**0**	**0**	**0**	**0**	**0**	**0**	**0**	**0**	**0**	**0**	**0**	**0**	**2**	**2**	**4**	**6**	**23**

[a] Score = 0-3, no intervention. Score = ≥ 4 (or ≥ 3 in any single parameter), additional monitoring of at least once in the evening 4-6 hours after the final late afternoon check. Score

[1] Consecutive days with NO biscuit consumption only.

[2] Consecutive days with NO enrichment consumption only.

A=As applicable

Clinical Scoring: MARV-exposed NHP 8

Parameter	Degree of Parameter	Score[a]	Score by Study Day																											
			-4		-3		-2		-1		0		1		2		3		4		5		6			7			8
			Ob 1	Ob 2	Ob 1	Ob 2	Ob 1	Ob 2	Ob 1	Ob 2	Ob 1	Ob 2	Ob 1	Ob 2	Ob 1	Ob 2	Ob 1	Ob 2	Ob 1	Ob 2	Ob 1	Ob 2	Ob 1	Ob 2	Ob 3	Ob 1	Ob 2	Ob 3	Ob 1
Respiration	Normal	0	0	0	0	0	0	0	0	0	0	0	0	0	0	0	0	0	0	0	0	0	0	0	0	0	0		
	Abdominal breathing or labored breathing	4																										4	
	Severe dyspnea; agonal breathing	10																											10
Food Consumption Feces/Urine	Normal	0	0		0		0		0		0		0		0		0		0		0		0			0			NS
	No biscuits eaten	1																											
	Consecutive days (Day 2=2, Day 3=3, Day 4=4, etc.)[1]	A																											
	No enrichment eaten	1																											
	Consecutive days (Day 2=3, Day 3=4, Day 4=5, etc.)[2]	A																											
	No feces seen (AM check); no urine seen (AM check)	1																											
	Diarrhea (liquid)	2																											
Activity/ Appearance	Normal	0	0	0	0	0	0	0	0	0	0	0	0	0	0	0	0	0	0	0	0	0	0	0	0				
	Hunched but active most of the time	1																								1			
	Hunched with head between knees; dull appearance to eyes	3																									3	3	
	Lies down; gets up when approached	4																											
	Lies down; gets up with some prodding but not when approached	10																											10
Bleeding/ Hemorrhage	No Signs	0	0	0	0	0	0	0	0	0	0	0	0	0	0	0	0	0	0	0	0	0	0	0	0				
	Petechiation and/or ecchymosis	2																								2	2	2	2
	Observable bleeding; controlled by clotting (not menses) AND/OR Petechiation and/or ecchymosis > 50%	4																											
	Uncontrolled Bleeding	10																											
		**Total Score**	**0**	**0**	**0**	**0**	**0**	**0**	**0**	**0**	**0**	**0**	**0**	**0**	**0**	**0**	**0**	**0**	**0**	**0**	**0**	**0**	**0**	**0**	**0**	**3**	**5**	**9**	**22**

[a] Score = 0-3, no intervention. Score = ≥ 4 (or ≥ 3 in any single parameter), additional monitoring of at least once in the evening 4-6 hours after the final late afternoon check. Score

[1] Consecutive days with NO biscuit consumption only.

[2] Consecutive days with NO enrichment consumption only.

A=As applicable

NS = No Score recorded

Clinical Scoring: MARV-exposed NHP 9																											
Parameter	Degree of Parameter	Score[a]	Score by Study Day																								
			-4		-3		-2		-1		0		1		2		3		4		5		6			7	
			Ob 1	Ob 2	Ob 1	Ob 2	Ob 1	Ob 2	Ob 1	Ob 2	Ob 1	Ob 2	Ob 1	Ob 2	Ob 1	Ob 2	Ob 1	Ob 2	Ob 1	Ob 2	Ob 1	Ob 2	Ob 1	Ob 2	Ob 3	Ob 1	Ob 2
Respiration	Normal	0	0	0	0	0	0	0	0	0	0	0	0	0	0	0	0	0	0	0	0	0	0	0	0	0	
	Abdominal breathing or labored breathing	4																									4
	Severe dyspnea; agonal breathing	10																									
Food Consumption Feces/Urine	Normal	0	0		0		0		0		0		0		0		0		0		0						
	No biscuits eaten	1																					1	1	1		
	Consecutive days (Day 2=2, Day 3=3, Day 4=4, etc.)[1]	A																								2	2
	No enrichment eaten	1																									
	Consecutive days (Day 2=3, Day 3=4, Day 4=5, etc.)[2]	A																									
	No feces seen (AM check); no urine seen (AM check)	1																									
	Diarrhea (liquid)	2																									
Activity/ Appearance	Normal	0	0	0	0	0	0	0	0	0	0	0	0	0	0	0	0	0	0	0	0	0	0	0	0		
	Hunched but active most of the time	1																									
	Hunched with head between knees; dull appearance to eyes	3																								3	3
	Lies down; gets up when approached	4																									
	Lies down; gets up with some prodding but not when approached	10																									
Bleeding/ Hemorrhage	No Signs	0	0	0	0	0	0	0	0	0	0	0	0	0	0	0	0	0	0	0	0	0	0				
	Petechiation and/or ecchymosis	2																						2	2	2	2
	Observable bleeding; controlled by clotting (not menses) AND/OR Petechiation and/or ecchymosis > 50%	4																									
	Uncontrolled Bleeding	10																									
		**Total Score**	**0**	**0**	**0**	**0**	**0**	**0**	**0**	**0**	**0**	**0**	**0**	**0**	**0**	**0**	**0**	**0**	**0**	**0**	**0**	**0**	**1**	**3**	**3**	**7**	**11**

[a] Score = 0-3, no intervention. Score = ≥ 4 (or ≥ 3 in any single parameter), additional monitoring of at least once in the evening 4-6 hours after the final late afternoon check. Score

[1] Consecutive days with NO biscuit consumption only.

[2] Consecutive days with NO enrichment consumption only.

A=As applicable

Clinical Scoring: MARV-exposed NHP 10																														
Parameter	Degree of Parameter	Score[a]	Score by Study Day																										
			-4		-3		-2		-1		0		1		2		3		4		5		6			7			8
			Ob 1	Ob 2	Ob 1	Ob 2	Ob 1	Ob 2	Ob 1	Ob 2	Ob 1	Ob 2	Ob 1	Ob 2	Ob 1	Ob 2	Ob 1	Ob 2	Ob 1	Ob 2	Ob 1	Ob 2	Ob 1	Ob 2	Ob 3	Ob 1	Ob 2	Ob 3	Ob 1
Respiration	Normal	0	0	0	0	0	0	0	0	0	0	0	0	0	0	0	0	0	0	0	0	0	0	0	0	0	0	0	
	Abdominal breathing or labored breathing	4																											4
	Severe dyspnea; agonal breathing	10																											
Food Consumption Feces/Urine	Normal	0	0		0		0		0		0		0		0		0		0		0								
	No biscuits eaten	1																					1	1	1				
	Consecutive days (Day 2=2, Day 3=3, Day 4=4, etc.)[1]	A																								2	2	2	
	No enrichment eaten	1																											3
	Consecutive days (Day 2=3, Day 3=4, Day 4=5, etc.)[2]	A																											
	No feces seen (AM check); no urine seen (AM check)	1																											
	Diarrhea (liquid)	2																											
Activity/ Appearance	Normal	0	0	0	0	0	0	0	0	0	0	0	0	0	0	0	0	0	0	0	0	0	0	0	0	0	0		
	Hunched but active most of the time	1																										1	
	Hunched with head between knees; dull appearance to eyes	3																											
	Lies down; gets up when approached	4																											
	Lies down; gets up with some prodding but not when approached	10																											10
Bleeding/ Hemorrhage	No Signs	0	0	0	0	0	0	0	0	0	0	0	0	0	0	0	0	0	0	0	0	0	0	0	0				
	Petechiation and/or ecchymosis	2																								2	2	2	2
	Observable bleeding; controlled by clotting (not menses) AND/OR Petechiation and/or ecchymosis > 50%	4																											
	Uncontrolled Bleeding	10																											
		**Total Score**	**0**	**0**	**0**	**0**	**0**	**0**	**0**	**0**	**0**	**0**	**0**	**0**	**0**	**0**	**0**	**0**	**0**	**0**	**0**	**0**	**1**	**1**	**1**	**4**	**4**	**5**	**19**

[a] Score = 0-3, no intervention. Score = ≥ 4 (or ≥ 3 in any single parameter), additional monitoring of at least once in the evening 4-6 hours after the final late afternoon check. Score

[1] Consecutive days with NO biscuit consumption only.

[2] Consecutive days with NO enrichment consumption only.

A=As applicable

Clinical Scoring: MARV-exposed NHP 11																										
Parameter	Degree of Parameter	Score[a]	Score by Study Day																							
			-4		-3		-2		-1		0		1		2		3		4		5		6			7
			Ob 1	Ob 2	Ob 1	Ob 2	Ob 1	Ob 2	Ob 1	Ob 2	Ob 1	Ob 2	Ob 1	Ob 2	Ob 1	Ob 2	Ob 1	Ob 2	Ob 1	Ob 2	Ob 1	Ob 2	Ob 1	Ob 2	Ob 3	Ob 1
Respiration	Normal	0	0	0	0	0	0	0	0	0	0	0	0	0	0	0	0	0	0	0	0	0	0	0	0	NS
	Abdominal breathing or labored breathing	4																								
	Severe dyspnea; agonal breathing	10																								
Food Consumption Feces/Urine	Normal	0	0		0		0		0		0		0		0		0		0		0					NS
	No biscuits eaten	1																					1	1	1	
	Consecutive days (Day 2=2, Day 3=3, Day 4=4, etc.)[1]	A																								
	No enrichment eaten	1																								
	Consecutive days (Day 2=3, Day 3=4, Day 4=5, etc.) [2]	A																								
	No feces seen (AM check); no urine seen (AM check)	1																								
	Diarrhea (liquid)	2																								
Activity/ Appearance	Normal	0	0	0	0	0	0	0	0	0	0	0	0	0	0	0	0	0	0	0	0	0	0	0	0	NS
	Hunched but active most of the time	1																								
	Hunched with head between knees; dull appearance to eyes	3																								
	Lies down; gets up when approached	4																								
	Lies down; gets up with some prodding but not when approached	10																								
Bleeding/ Hemorrhage	No Signs	0	0	0	0	0	0	0	0	0	0	0	0	0	0	0	0	0	0	0						NS
	Petechiation and/or ecchymosis	2																			2	2	2	2	2	
	Observable bleeding; controlled by clotting (not menses) AND/OR Petechiation and/or ecchymosis > 50%	4																								
	Uncontrolled Bleeding	10																								
**Total Score**			**0**	**0**	**0**	**0**	**0**	**0**	**0**	**0**	**0**	**0**	**0**	**0**	**0**	**0**	**0**	**0**	**0**	**0**	**2**	**2**	**3**	**3**	**3**	**FD**

[a] Score = 0-3, no intervention. Score = ≥ 4 (or ≥ 3 in any single parameter), additional monitoring of at least once in the evening 4-6 hours after the final late afternoon check. Score

[1] Consecutive days with NO biscuit consumption only.

[2] Consecutive days with NO enrichment consumption only.

A=As applicable

NS = No Score Recorded, FD

FD = Found Dead

Clinical Scoring: MARV-exposed NHP 12																										
Parameter	Degree of Parameter	Score[a]	Score by Study Day																							
			-4		-3		-2		-1		0		1		2		3		4		5		6			7
			Ob 1	Ob 2	Ob 1	Ob 2	Ob 1	Ob 2	Ob 1	Ob 2	Ob 1	Ob 2	Ob 1	Ob 2	Ob 1	Ob 2	Ob 1	Ob 2	Ob 1	Ob 2	Ob 1	Ob 2	Ob 1	Ob 2	Ob 3	Ob 1
Respiration	Normal	0	0	0	0	0	0	0	0	0	0	0	0	0	0	0	0	0	0	0	0	0	0	0	0	
	Abdominal breathing or labored breathing	4																								4
	Severe dyspnea; agonal breathing	10																								
Food Consumption Feces/Urine	Normal	0	0		0		0		0		0		0		0		0		0							
	No biscuits eaten	1																			1	1				
	Consecutive days (Day 2=2, Day 3=3, Day 4=4, etc.)[1]	A																					2	2	2	3
	No enrichment eaten	1																								
	Consecutive days (Day 2=3, Day 3=4, Day 4=5, etc.) [2]	A																								
	No feces seen (AM check); no urine seen (AM check)	1																								
	Diarrhea (liquid)	2																								
Activity/ Appearance	Normal	0	0	0	0	0	0	0	0	0	0	0	0	0	0	0	0	0	0	0	0	0	0	0	0	
	Hunched but active most of the time	1																								
	Hunched with head between knees; dull appearance to eyes	3																								
	Lies down; gets up when approached	4																								
	Lies down; gets up with some prodding but not when approached	10																								10
Bleeding/ Hemorrhage	No Signs	0	0	0	0	0	0	0	0	0	0	0	0	0	0	0	0	0	0	0						
	Petechiation and/or ecchymosis	2																			2	2	2	2	2	2
	Observable bleeding; controlled by clotting (not menses) AND/OR Petechiation and/or ecchymosis > 50%	4																								
	Uncontrolled Bleeding	10																								
		**Total Score**	**0**	**0**	**0**	**0**	**0**	**0**	**0**	**0**	**0**	**0**	**0**	**0**	**0**	**0**	**0**	**0**	**0**	**0**	**3**	**3**	**4**	**4**	**4**	**19**

[a] Score = 0-3, no intervention. Score = ≥ 4 (or ≥ 3 in any single parameter), additional monitoring of at least once in the evening 4-6 hours after the final late afternoon check. Score ≥ 10, Euthanasia.

[1] Consecutive days with NO biscuit consumption only.

[2] Consecutive days with NO enrichment consumption only.

A=As applicable
