# Supplementary material for: Natural History of Marburg Virus Infection to Support Medical Countermeasure Development
Source: Viruses. 2022 Oct 18;14(10):2291. doi: 10.3390/v14102291 (PMC9607268; doi:10.3390/v14102291)
Supplement: Supplementary file 1 [file viruses-14-02291-s001.zip › Table S5 Mock Exposed Clinical Scores.pdf]

Table S5. Clinical Scoring for Mock-exposed NHPs

Clinical Scoring: Mock-exposed NHP 1

Parameter	Degree of Parameter	Score[a]	Score by Study Day																														
			-4		-3		-2		-1		0		1		2		3		4		5		6			7			8		9		10-28
			Ob 1	Ob 2	Ob 1	Ob 2	Ob 1	Ob 2	Ob 1	Ob 2	Ob 1	Ob 2	Ob 1	Ob 2	Ob 1	Ob 2	Ob 1	Ob 2	Ob 1	Ob 2	Ob 1	Ob 2	Ob 1	Ob 2	Ob 3	Ob 1	Ob 2	Ob 3	Ob 1	Ob 2	Ob 1	Ob 2	Ob 1
Respiration	Normal	0	0	0	0	0	0	0	0	0	0	0	0	0	0	0	0	0	0	0	0	0	0	0	0	0	0	0	0	0	0	0	0
	Abdominal breathing or labored breathing	4																															
	Severe dyspnea; agonal breathing	10																															
Food Consumption Feces/Urine	Normal	0	0		0		0		0		0		0		0		0		0		0		0			0			0		0		0
	No biscuits eaten	1																															
	Consecutive days (Day 2=2, Day 3=3, Day 4=4, etc.)[1]	A																															
	No enrichment eaten	1																															
	Consecutive days (Day 2=3, Day 3=4, Day 4=5, etc.)[2]	A																															
	No feces seen (AM check); no urine seen (AM check)	1																															
	Diarrhea (liquid)	2																															
Activity/ Appearance	Normal	0	0	0	0	0	0	0	0	0	0	0	0	0	0	0	0	0	0	0	0	0	0	0	0	0	0	0	0	0	0	0	0
	Hunched but active most of the time	1																															
	Hunched with head between knees; dull appearance to eyes	3																															
	Lies down; gets up when approached	4																															
	Lies down; gets up with some prodding but not when approached	10																															
Bleeding/ Hemorrhage	No Signs	0	0	0	0	0	0	0	0	0	0	0	0	0	0	0	0	0	0	0	0	0	0	0	0	0	0	0	0	0	0	0	0
	Petechiation and/or ecchymosis	2																															
	Observable bleeding; controlled by clotting (not menses) AND/OR Petechiation and/or ecchymosis > 50%	4																															
	Uncontrolled Bleeding	10																															
	**Total Score**		**0**	**0**	**0**	**0**	**0**	**0**	**0**	**0**	**0**	**0**	**0**	**0**	**0**	**0**	**0**	**0**	**0**	**0**	**0**	**0**	**0**	**0**	**0**	**0**	**0**	**0**	**0**	**0**	**0**	**0**	**0**

[a] Score = 0-3, no intervention. Score = ≥ 4 (or ≥ 3 in any single parameter), additional monitoring of at least once in the evening 4-6 hours after the final late afternoon check. Score ≥ 10, Euthanasia.

[1] Consecutive days with NO biscuit consumption only.

[2] Consecutive days with NO enrichment consumption only.

A=As applicable

Clinical Scoring: Mock-exposed NHP 2																																	
**Parameter**	**Degree of Parameter**	**Score[a]**	**Score by Study Day**																														
			-4		-3		-2		-1		0		1		2		3		4		5		6			7			8		9		10-28
			**Ob 1**	**Ob 2**	**Ob 1**	**Ob 2**	**Ob 1**	**Ob 2**	**Ob 1**	**Ob 2**	**Ob 1**	**Ob 2**	**Ob 1**	**Ob 2**	**Ob 1**	**Ob 2**	**Ob 1**	**Ob 2**	**Ob 1**	**Ob 2**	**Ob 1**	**Ob 2**	**Ob 1**	**Ob 2**	**Ob 3**	**Ob 1**	**Ob 2**	**Ob 3**	**Ob 1**	**Ob 2**	**Ob 1**	**Ob 2**	**Ob 1**
**Respiration**	Normal	0	0	0	0	0	0	0	0	0	0	0	0	0	0	0	0	0	0	0	0	0	0	0	0	0	0	0	0	0	0	0	0
	Abdominal breathing or labored breathing	4																															
	Severe dyspnea; agonal breathing	10																															
**Food Consumption Feces/Urine**	Normal	0	0		0		0		0		0		0		0		0		0		0		0			0			0		0		0
	No biscuits eaten	1																															
	Consecutive days (Day 2=2, Day 3=3, Day 4=4, etc.)[1]	A																															
	No enrichment eaten	1																															
	Consecutive days (Day 2=3, Day 3=4, Day 4=5, etc.)[2]	A																															
	No feces seen (AM check); no urine seen (AM check)	1																															
	Diarrhea (liquid)	2																															
**Activity/ Appearance**	Normal	0	0	0	0	0	0	0	0	0	0	0	0	0	0	0	0	0	0	0	0	0	0	0	0	0	0	0	0	0	0	0	0
	Hunched but active most of the time	1																															
	Hunched with head between knees; dull appearance to eyes	3																															
	Lies down; gets up when approached	4																															
	Lies down; gets up with some prodding but not when approached	10																															
**Bleeding/ Hemorrhage**	No Signs	0	0	0	0	0	0	0	0	0	0	0	0	0	0	0	0	0	0	0	0	0	0	0	0	0	0	0	0	0	0	0	0
	Petechiation and/or ecchymosis	2																															
	Observable bleeding; controlled by clotting (not menses) AND/OR Petechiation and/or ecchymosis > 50%	4																															
	Uncontrolled Bleeding	10																															
		**Total Score**	**0**	**0**	**0**	**0**	**0**	**0**	**0**	**0**	**0**	**0**	**0**	**0**	**0**	**0**	**0**	**0**	**0**	**0**	**0**	**0**	**0**	**0**	**0**	**0**	**0**	**0**	**0**	**0**	**0**	**0**	**0**

[a] Score = 0-3, no intervention. Score = ≥ 4 (or ≥ 3 in any single parameter), additional monitoring of at least once in the evening 4-6 hours after the final late afternoon check. Score ≥ 10, Euthanasia.

[1] Consecutive days with NO biscuit consumption only.

[2] Consecutive days with NO enrichment consumption only.

A=As applicable

Clinical Scoring: Mock-exposed NHP 3

Parameter	Degree of Parameter	Score[a]	Score by Study Day																														
			-4		-3		-2		-1		0		1		2		3		4		5		6			7			8		9		10-28
			Ob 1	Ob 2	Ob 1	Ob 2	Ob 1	Ob 2	Ob 1	Ob 2	Ob 1	Ob 2	Ob 1	Ob 2	Ob 1	Ob 2	Ob 1	Ob 2	Ob 1	Ob 2	Ob 1	Ob 2	Ob 1	Ob 2	Ob 3	Ob 1	Ob 2	Ob 3	Ob 1	Ob 2	Ob 1	Ob 2	Ob 1
Respiration	Normal	0	0	0	0	0	0	0	0	0	0	0	0	0	0	0	0	0	0	0	0	0	0	0	0	0	0	0	0	0	0	0	0
	Abdominal breathing or labored breathing	4																															
	Severe dyspnea; agonal breathing	10																															
Food Consumption Feces/Urine	Normal	0	0		0		0		0		0		0		0		0		0		0		0			0			0		0		0
	No biscuits eaten	1																															
	Consecutive days (Day 2=2, Day 3=3, Day 4=4, etc.)[1]	A																															
	No enrichment eaten	1																															
	Consecutive days (Day 2=3, Day 3=4, Day 4=5, etc.)[2]	A																															
	No feces seen (AM check); no urine seen (AM check)	1																															
	Diarrhea (liquid)	2																															
Activity/ Appearance	Normal	0	0	0	0	0	0	0	0	0	0	0	0	0	0	0	0	0	0	0	0	0	0	0	0	0	0	0	0	0	0	0	0
	Hunched but active most of the time	1																															
	Hunched with head between knees; dull appearance to eyes	3																															
	Lies down; gets up when approached	4																															
	Lies down; gets up with some prodding but not when approached	10																															
Bleeding/ Hemorrhage	No Signs	0	0	0	0	0	0	0	0	0	0	0	0	0	0	0	0	0	0	0	0	0	0	0	0	0	0	0	0	0	0	0	0
	Petechiation and/or ecchymosis	2																															
	Observable bleeding; controlled by clotting (not menses) AND/OR Petechiation and/or ecchymosis > 50%	4																															
	Uncontrolled Bleeding	10																															
	**Total Score**		**0**	**0**	**0**	**0**	**0**	**0**	**0**	**0**	**0**	**0**	**0**	**0**	**0**	**0**	**0**	**0**	**0**	**0**	**0**	**0**	**0**	**0**	**0**	**0**	**0**	**0**	**0**	**0**	**0**	**0**	**0**

[a] Score = 0-3, no intervention. Score = ≥ 4 (or ≥ 3 in any single parameter), additional monitoring of at least once in the evening 4-6 hours after the final late afternoon check. Score ≥ 10, Euthanasia.

[1] Consecutive days with NO biscuit consumption only.

[2] Consecutive days with NO enrichment consumption only.

A=As applicable

Clinical Scoring: Mock-exposed NHP 4

Parameter	Degree of Parameter	Score[a]	Score by Study Day																														
			-4		-3		-2		-1		0		1		2		3		4		5		6			7			8		9		10-28
			**Ob 1**	**Ob 2**	**Ob 1**	**Ob 2**	**Ob 1**	**Ob 2**	**Ob 1**	**Ob 2**	**Ob 1**	**Ob 2**	**Ob 1**	**Ob 2**	**Ob 1**	**Ob 2**	**Ob 1**	**Ob 2**	**Ob 1**	**Ob 2**	**Ob 1**	**Ob 2**	**Ob 1**	**Ob 2**	**Ob 3**	**Ob 1**	**Ob 2**	**Ob 3**	**Ob 1**	**Ob 2**	**Ob 1**	**Ob 2**	**Ob 1**
**Respiration**	Normal	0	0	0	0	0	0	0	0	0	0	0	0	0	0	0	0	0	0	0	0	0	0	0	0	0	0	0	0	0	0	0	0
	Abdominal breathing or labored breathing	4																															
	Severe dyspnea; agonal breathing	10																															
**Food Consumption Feces/Urine**	Normal	0	0		0		0		0		0		0		0		0		0		0		0			0			0		0		0
	No biscuits eaten	1																															
	Consecutive days (Day 2=2, Day 3=3, Day 4=4, etc.)[1]	A																															
	No enrichment eaten	1																															
	Consecutive days (Day 2=3, Day 3=4, Day 4=5, etc.)[2]	A																															
	No feces seen (AM check); no urine seen (AM check)	1																															
	Diarrhea (liquid)	2																															
**Activity/ Appearance**	Normal	0	0	0	0	0	0	0	0	0	0	0	0	0	0	0	0	0	0	0	0	0	0	0	0	0	0	0	0	0	0	0	0
	Hunched but active most of the time	1																															
	Hunched with head between knees; dull appearance to eyes	3																															
	Lies down; gets up when approached	4																															
	Lies down; gets up with some prodding but not when approached	10																															
**Bleeding/ Hemorrhage**	No Signs	0	0	0	0	0	0	0	0	0	0	0	0	0	0	0	0	0	0	0	0	0	0	0	0	0	0	0	0	0	0	0	0
	Petechiation and/or ecchymosis	2																															
	Observable bleeding; controlled by clotting (not menses) AND/OR Petechiation and/or ecchymosis > 50%	4																															
	Uncontrolled Bleeding	10																															
		**Total Score**	**0**	**0**	**0**	**0**	**0**	**0**	**0**	**0**	**0**	**0**	**0**	**0**	**0**	**0**	**0**	**0**	**0**	**0**	**0**	**0**	**0**	**0**	**0**	**0**	**0**	**0**	**0**	**0**	**0**	**0**	**0**

[a] Score = 0-3, no intervention. Score = ≥ 4 (or ≥ 3 in any single parameter), additional monitoring of at least once in the evening 4-6 hours after the final late afternoon check. Score ≥ 10, Euthanasia.

[1] Consecutive days with NO biscuit consumption only.

[2] Consecutive days with NO enrichment consumption only.

A=As applicable

Clinical Scoring: Mock-exposed NHP 5

Parameter	Degree of Parameter	Score[a]	Score by Study Day																														
			-4		-3		-2		-1		0		1		2		3		4		5		6			7			8		9		10-28
			Ob 1	Ob 2	Ob 1	Ob 2	Ob 1	Ob 2	Ob 1	Ob 2	Ob 1	Ob 2	Ob 1	Ob 2	Ob 1	Ob 2	Ob 1	Ob 2	Ob 1	Ob 2	Ob 1	Ob 2	Ob 1	Ob 2	Ob 3	Ob 1	Ob 2	Ob 3	Ob 1	Ob 2	Ob 1	Ob 2	Ob 1
**Respiration**	Normal	0	0	0	0	0	0	0	0	0	0	0	0	0	0	0	0	0	0	0	0	0	0	0	0	0	0	0	0	0	0	0	0
	Abdominal breathing or labored breathing	4																															
	Severe dyspnea; agonal breathing	10																															
**Food Consumption Feces/Urine**	Normal	0	0		0		0		0		0		0		0		0		0		0		0			0			0		0		0
	No biscuits eaten	1																															
	Consecutive days (Day 2=2, Day 3=3, Day 4=4, etc.)[1]	A																															
	No enrichment eaten	1																															
	Consecutive days (Day 2=3, Day 3=4, Day 4=5, etc.)[2]	A																															
	No feces seen (AM check); no urine seen (AM check)	1																															
	Diarrhea (liquid)	2																															
**Activity/ Appearance**	Normal	0	0	0	0	0	0	0	0	0	0	0	0	0	0	0	0	0	0	0	0	0	0	0	0	0	0	0	0	0	0	0	0
	Hunched but active most of the time	1																															
	Hunched with head between knees; dull appearance to eyes	3																															
	Lies down; gets up when approached	4																															
	Lies down; gets up with some prodding but not when approached	10																															
**Bleeding/ Hemorrhage**	No Signs	0	0	0	0	0	0	0	0	0	0	0	0	0	0	0	0	0	0	0	0	0	0	0	0	0	0	0	0	0	0	0	0
	Petechiation and/or ecchymosis	2																															
	Observable bleeding; controlled by clotting (not menses) AND/OR Petechiation and/or ecchymosis > 50%	4																															
	Uncontrolled Bleeding	10																															
		**Total Score**	**0**	**0**	**0**	**0**	**0**	**0**	**0**	**0**	**0**	**0**	**0**	**0**	**0**	**0**	**0**	**0**	**0**	**0**	**0**	**0**	**0**	**0**	**0**	**0**	**0**	**0**	**0**	**0**	**0**	**0**	**0**

[a] Score = 0-3, no intervention. Score = ≥ 4 (or ≥ 3 in any single parameter), additional monitoring of at least once in the evening 4-6 hours after the final late afternoon check. Score ≥ 10, Euthanasia.

[1] Consecutive days with NO biscuit consumption only.

[2] Consecutive days with NO enrichment consumption only.

A=As applicable

Clinical Scoring: Mock-exposed NHP 6

Parameter	Degree of Parameter	Score[a]	Score by Study Day																														
			-4		-3		-2		-1		0		1		2		3		4		5		6			7			8		9		10-28
			Ob 1	Ob 2	Ob 1	Ob 2	Ob 1	Ob 2	Ob 1	Ob 2	Ob 1	Ob 2	Ob 1	Ob 2	Ob 1	Ob 2	Ob 1	Ob 2	Ob 1	Ob 2	Ob 1	Ob 2	Ob 1	Ob 2	Ob 3	Ob 1	Ob 2	Ob 3	Ob 1	Ob 2	Ob 1	Ob 2	Ob 1
Respiration	Normal	0	0	0	0	0	0	0	0	0	0	0	0	0	0	0	0	0	0	0	0	0	0	0	0	0	0	0	0	0	0	0	0	0	0
	Abdominal breathing or labored breathing	4																																	
	Severe dyspnea; agonal breathing	10																																	
Food Consumption Feces/Urine	Normal	0	0		0		0		0		0		0		0		0		0		0		0			0			0		0		0
	No biscuits eaten	1																																	
	Consecutive days (Day 2=2, Day 3=3, Day 4=4, etc.)[1]	A																																	
	No enrichment eaten	1																																	
	Consecutive days (Day 2=3, Day 3=4, Day 4=5, etc.)[2]	A																																	
	No feces seen (AM check); no urine seen (AM check)	1																																	
	Diarrhea (liquid)	2																																	
Activity/ Appearance	Normal	0	0	0	0	0	0	0	0	0	0	0	0	0	0	0	0	0	0	0	0	0	0	0	0	0	0	0	0	0	0	0	0	0	0
	Hunched but active most of the time	1																																	
	Hunched with head between knees; dull appearance to eyes	3																																	
	Lies down; gets up when approached	4																																	
	Lies down; gets up with some prodding but not when approached	10																																	
Bleeding/ Hemorrhage	No Signs	0	0	0	0	0	0	0	0	0	0	0	0	0	0	0	0	0	0	0	0	0	0	0	0	0	0	0	0	0	0	0	0	0	0
	Petechiation and/or ecchymosis	2																																	
	Observable bleeding; controlled by clotting (not menses) AND/OR Petechiation and/or ecchymosis > 50%	4																																	
	Uncontrolled Bleeding	10																																	
		**Total Score**	**0**	**0**	**0**	**0**	**0**	**0**	**0**	**0**	**0**	**0**	**0**	**0**	**0**	**0**	**0**	**0**	**0**	**0**	**0**	**0**	**0**	**0**	**0**	**0**	**0**	**0**	**0**	**0**	**0**	**0**	**0**	**0**	**0**	**0**

[a] Score = 0-3, no intervention. Score = ≥ 4 (or ≥ 3 in any single parameter), additional monitoring of at least once in the evening 4-6 hours after the final late afternoon check. Score ≥ 10, Euthanasia.

[1] Consecutive days with NO biscuit consumption only.

[2] Consecutive days with NO enrichment consumption only.

A=As applicable
